# Mining the Gaps: Rethinking Divergence Between Biological and Self‐Report Measures in the Study of Sexual Diversity

**DOI:** 10.1002/ajhb.70201

**Published:** 2026-01-21

**Authors:** Lisa M. Diamond

**Affiliations:** ^1^ Department of Psychology University of Utah Salt Lake City Utah USA

**Keywords:** biology, genetics, self‐report, sexual arousal, sexual orientation

## Abstract

Over the past several decades, scholars have conducted hundreds of studies investigating potential biomarkers of sexual orientation, such as genes, neuroanatomical features, and patterns of physiological response to sexual stimuli. The findings have been inconsistent: Biological measures sometimes converge with—but just as often *diverge from*—individuals' self‐reported sexual attractions, behaviors, and identities. For example, numerous studies show that individuals' biological responses to erotic stimuli frequently diverge from their self‐reported sexual identities *and* self‐reported arousal to such stimuli. I argue that such cases of “biodivergence” warrant a shift in our conceptualization of sexual orientation, from seeing it as a singular and coherent phenotype to seeing it as a constellation of *multiple* biobehavioral patterns, with multiple and divergent causes and effects. I show that this perspective concords with recent findings from genetic research on sexual orientation, which show there is no single genotype underlying patterns of same‐gender expression, and also concords with recent population data showing increasingly varied and fluid forms of sexual diversity around the globe that challenge the notion of sexual orientation as a singular and coherent sexual phenotype.

## 1 Introduction

The use of biological measures to study sexual orientation has a long history (reviewed in Jordan‐Young 2011), but surged markedly in the 1990s, paralleling similar trends in other domains of psychology after the US Congress proclaimed the 1990s “The Decade of the Brain” (United States Congress 1990). During this period, some psychologists expected the use of biological measures to revolutionize “the unraveling of the mind's knots and nightmares” (Cacioppo and Berntson 1992, 1020). Sexual orientation is arguably among the mind's most stubborn “knots,” and so it is not surprising that psychologists increasingly applied biological measures to its unraveling. Specifically, researchers have sought to identify biological processes or indicators*—*genes, neuroanatomical features, patterns of hormonal exposure or physiological response—that could reliably distinguish between heterosexual and nonheterosexual individuals (Attard‐Johnson et al. 2021; Huberman and Chivers 2015; LeVay 2011; Prantner et al. 2025).

Feminist bioscientists have critiqued such studies for their implicit adherence to *biological determinism* (the assumption that human experiences are primarily caused by biological factors) and *biological reductionism* (the assumption that human experiences are best understood by reducing them to their biological underpinnings, Jordan‐Young 2011; van Anders 2024). Yet the findings of these studies subvert *both* of these “isms,” because no reliable biomarkers of sexual orientation have been found, despite three decades of effort. The most accurate summary of the collected findings is that they are consistently inconsistent: sometimes we detect biological differences between self‐identified heterosexual and nonheterosexual individuals, and sometimes we don't.

By far the most striking example concerns studies of sexual arousal, the very experience thought to define an individual's sexual orientation. Experimental assessments have found that individuals' biological responses to same‐gender and other‐gender erotic videos often diverge from individuals' self‐reported sexual identities *and* their self‐reported arousal to these videos. In this paper I review these puzzling findings of “biodivergence” to support a shift in our conceptualization of sexual orientation, from a singular and coherent trait to a constellation of multiple and divergent biobehavioral patterns, with multiple and divergent causes and effects (for related views, see Diamond 2023; van Anders 2015). I argue that this conceptual shift can help account for one of the most perplexing findings from genetic research on sexual orientation: that there are not only multiple genes, but multiple *genotypes* associated with patterns of same‐gender expression. Reconceptualizing sexual orientation as multiple and divergent biobehavioral patterns also concords with the increasingly varied and fluid forms of sexual and gender diversity now documented around the world and in virtual spaces (reviewed in Hammack and Wignall 2023), which are difficult to reconcile with conventional trait models of sexual orientation. Reviewing these findings, Hammack (2025) recently called for a shift in our approach to studying sexual diversity, away from studying “types of people” and toward studying types of *phenomena*. Although Hammack was focusing on behavioral and cultural enactments of sexual/gender diversity, I would argue that the same shift—from a search for coherent categories to a synthesis of multiple and divergent phenomena—is equally critical for biological research on sexual/gender diversity.

### 1.1 Biology Versus Self‐Report: Which Is Right?

Let's begin with a basic question: Why use biological measures *at all* to study sexual orientation? Historically, the implicit answer has been “Because they don't lie.” The only way to assess an individual's sexual orientation is to *ask them*, but researchers have long acknowledged that individuals may not accurately report sexual feelings and behaviors that are socially stigmatized (Boyer et al. 2012; Brody et al. 2003)? How, then, can we differentiate between individuals who are “authentically” queer (i.e., nonheterosexual) and those who might be confused, experimenting, curious, or opportunistic (Bell et al. 1981; Money 1987). Laypeople have made the same distinctions: Women who identify as queer in college, only to identify as heterosexual after graduating, have jokingly been called “LUGs” or “BUGs” (lesbian/bisexual until graduation), and often viewed as less authentic members of the queer community (reviewed in Diamond 2008). Parents, too, sometimes wonder whether a child who identifies as queer is *really* queer, or unduly influenced by peers (Davidson 2015; Lang 2022; Newman 2022; Rosky 2013), and numerous websites offer the prospect of “testing” one's sexual orientation with (unvalidated) online questionnaires (e.g., Curtis 2023; Schneider 2025).

Presumably, biological measures circumvent this problem by providing an index of sexual orientation that is independent of self‐report. The most widely used such measure is photoplethysmography (PPG), which quantifies changes in blood flow to the penis or vulva associated with sexual arousal. The historical use of PPG with sexual offenders provides a paradigmatic case of using biological measures to detect patterns of sexual arousal that individuals may not be willing to acknowledge (Jordan et al. 2019). Some scholars have gone so far as to call such measures “godsent gifts” to the study of sexual motivation, and have openly queried why self‐report measures remain in use at all (e.g., Ventura‐Aquino and Ågmo 2023, 10).

The notion that biological measures are more trustworthy measures of sexual orientation than self‐report was explicitly articulated in a controversial 2005 study seeking to validate the existence of bisexuality as a legitimate sexual orientation. At that time, many scholars and laypeople openly suspected that bisexuals were actually gay/lesbian individuals who were confused or closeted (reviewed in Rust 2000). Rieger and colleagues (Rieger et al. 2005) decided to test this hypothesis by experimentally comparing men's self‐reported sexual interest in men vs. women to their observed genital arousal (i.e., blood flow) while watching erotic videos of men and women. The study's findings appeared to confirm the authors' speculation that most bisexual men were “actually” gay or straight: One subgroup of bisexual men showed significantly more genital arousal to sexual stimuli featuring men versus women, despite *reporting* (with a continuous rating dial) comparable levels of subjective arousal to both genders. Another subgroup showed significantly more genital arousal to women than to men, but again reported comparable subjective arousal to both.

The authors offered only one potential explanation for the divergence between the genital and self‐report measures: One of them had to be wrong. As they speculated, “either bisexual men's subjective arousal has been *exaggerated* or their genital arousal has been *suppressed*” (Rieger et al. 2005 582, emphasis added). This conclusion is a paradigmatic example of *alignment normativity* (the assumption that alignment between different aspects of sexual phenomena is normative, and that misalignment indicates error or abnormality, van Anders 2024). Following the logic of biological reductionism, the authors openly privileged men's biological responses over their self‐reports, arguing that the latter were potentially distorted by bias, inaccuracy, or misrepresentation (a conclusion summarized in a New York Times article about the study as “Straight, Gay, or Lying,” Carey 2005). The authors therefore interpreted their results to indicate that bisexual orientations *might not exist* in men, despite bisexual men's own self‐perceptions.

Both laypeople and scientists criticized this conclusion as flawed and invalidating, and the study was eventually repeated with a more stringent set of inclusion criteria for bisexual men (specifically, a past history of sexual and romantic relationships with both men and women, Rosenthal et al. 2011, 2012). The second study *did* find evidence for bisexual patterns of genital arousal, and the authors concluded that their altered inclusion criteria may have successfully weeded out “men who might have adopted a bisexual identity for reasons other than attraction and arousal to both sexes” (Rosenthal et al. 2012, 145). Hence, although the second study overturned the prior study's claim about the nonexistence of male bisexuality, *it did so on the same biologically reductionist terms*, leaving unchallenged the assumption that biological indices of sexual arousal were more “correct” than individuals' self reports.

Neither study considered the possibility that *both* biological and self‐reported measures of sexual arousal were correct, but *different*, and that discrepancies between them were meaningful and informative in their own right. Yet this is the only possible conclusion from a groundbreaking series of studies of *women's* sexual arousal that definitively debunked the notion that sexual orientation could ever be biologically “diagnosed” in the laboratory.

### 1.2 Bio‐Divergence Diverges

In a groundbreaking series of studies stretching over two decades (Bouchard et al. 2015; Chivers et al. 2015, 2004, 2007; Chivers and Timmers 2012; Spape et al. 2014; Timmers et al. 2025), Chivers and colleagues examined individuals' genital and self‐reported arousal to erotic videos featuring their “preferred” versus “non‐preferred” targets (women represented the “preferred” target for lesbian‐identified women and heterosexual‐identified men, whereas men represented the “preferred” target for gay‐identified men and heterosexual‐identified women). The men in their study showed genital and self‐reported responses that matched their self‐identifications (i.e., greater genital and self‐reported arousal to erotic videos featuring their “preferred” versus “non‐preferred” gender). In short, gay men's genitals showed arousal to erotic videos featuring men, but not women; heterosexual men's genitals showed arousal to erotic videos featuring women, but not men.

Women, however, showed an altogether different pattern: Heterosexual women showed genital and self‐reported arousal to *both* women and men, a pattern Chivers denoted *nonspecificity*. Lesbian‐identified women showed more specificity than heterosexual women (i.e., greater genital and self‐reported arousal to stimuli featuring their preferred targets—women), but not quite as much specificity as men. Follow‐up studies using a range of different biological measures of sexual arousal (genital temperature, pupil dilation, eye movement, neural responses) confirmed these general patterns (Chivers and Blumenstock 2024), but also detected moderators, such as stimulus type (e.g., both heterosexual and queer women showed greater specificity in arousal when the experimental stimuli featured static images of aroused penises and vulvas as opposed to videos of sexual activity, Micanovic et al. 2021; Spape et al. 2014; Timmers et al. 2025).

The phenomenon of “nonspecificity” in women's genital arousal can be thought of as biodivergence at the *trait* level: i.e., patterns of biological arousal that diverge from the patterns presumed to characterize the “trait” of sexual orientation. Yet women in these studies also showed biodivergence at the *state* level: i.e., divergence between one's biological arousal to a sexual stimulus and one's concurrent self‐reported arousal to that stimulus. Specifically, cisgender women in these studies sometimes reported sexual arousal to a stimulus without showing corresponding increases in genital blood flow, or showed increased genital blood flow to a stimulus *without* reporting concurrent sexual arousal to that stimulus. This form of biodivergence has been denoted “nonconcordance.” Like “nonspecificity,” studies have consistently found this form of biodivergence to be more common among cisgender women than men, although varying in magnitude across different individuals and different studies (Chivers et al. 2010; Suschinsky and Lalumière 2012).

Both nonspecificity and nonconcordance challenge the conventional model of sexual orientation as a singular, coherent trait, *but for different reasons*. Nonspecificity (i.e., bio‐divergence at the *trait* level) challenges the expectation that sexual orientation manifests as a reliably “oriented” biological response to sexual stimuli. Nonconcordance (i.e., bio‐divergence at the *state* level) challenges the expectation that “arousal” is a coherent bio‐psychological experience to begin with. Accordingly, one might argue that the most important form of divergence captured by these phenomena is divergence between biological measures of sexual phenomena and our *expectations* that these measures should reflect individuals' self‐reported psychological experiences. Within the broader field of psychophysiological research, this assumption is denoted *isomorphism:* one‐to‐one correspondence between psychological states and biological processes. Yet as I review below, isomorphism is by no means the only way to conceptualize relations between psychological and biological processes, and consideration of alternative conceptualizations helps to show why findings of biodivergence in studies of sexual orientation should not be considered perplexing or erroneous, but a natural consequence of *multiplicity.*

### 1.3 Many‐To‐Many Instead of One‐To‐One

In an early and influential critique of psychologists' oversimplified reasoning about biological processes, Cacioppo and Tassinary argued that isomorphism represented a critical and common error in psychophysiological research, given that most psychological and biological processes are *multiply determined* (Cacioppo and Tassinary 1990). In fact, the findings of biodivergence that have emerged from psychophysiological studies of sexual arousal are matched by similar findings in psychophysiological studies of stress and other psychological states (Cacioppo et al. 2003; Mauss et al. 2005), which have been attributed to the inherent complexity of inferring complex, multiply‐determined psychological states (or traits) from single biomarkers. Sexual arousal, to be sure, is a paradigmatic example of a multiply‐determined state: Although we may *experience* sexual arousal as a coherent subjective experience (i.e., being “turned on”), it involves multiple biological processes operating across different time scales, including heightened activation of the sympathetic nervous system (manifested in respiratory changes, pupil dilation, cardiovascular activity), increased blood flow to, and engorgement of, multiple genital regions, increased production of hormones such as oxytocin, and increased activation of multiple brain regions, including the hypothalamus, the primary somatosensory cortex, the basal forebrain, and others (reviewed in Dickenson et al. 2020; Pfaus et al. 2014).

In such cases of complexity, Cacioppo and Tassinary (1990) urged psychophysiologists to consider four different patterns of biopsychological relations: *one‐to‐one* (i.e., a single psychological state associated with a single biomarker), *one‐to‐many* (a single psychological state associated with multiple biomarkers), *many‐to‐one* (multiple psychological states associated with a single biomarker), and *many‐to‐many* (multiple psychological states associated with multiple biomarkers). Not surprisingly, Cacioppo and Tassinary (1990) argued that most complex biopsychological phenomena involve *many‐to‐many* relations, which unfortunately yield the least reliable inferences and the greatest possibilities for misspecification.

**FIGURE 1:**
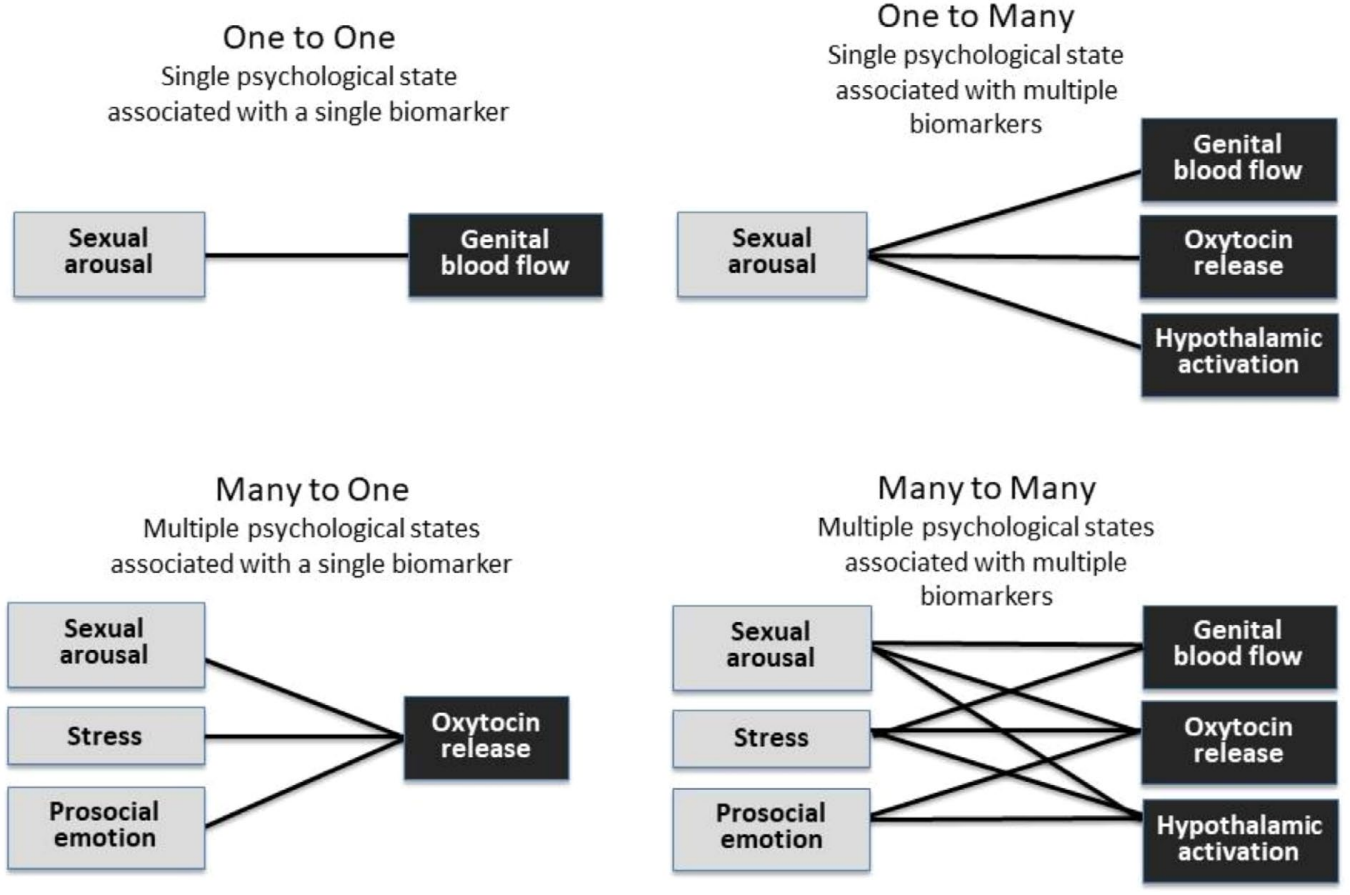
Four types of relations between psychological states related to sexual arousal and biomarkers of sexual arousal.

Figure 1 shows how Cacioppo and Tassinary's (1990) 4‐category framework might apply to the study of sexual arousal and orientation, and this figure helps to clarify why the expectation that biological measures of arousal would “signal” an individual's experience of arousal (and, in turn, their sexual orientation) was always oversimplified and unrealistic. The upper left quadrant depicts the form relationship that has been tested the most often in psychophysiological studies of sexual orientation: one‐to‐one associations between self‐reported sexual arousal and genital blood flow (PPG). Yet we cannot meaningfully interpret the presence *or* absence of such an association if there are *other* unspecified relations at play. Consider the upper right quadrant, which depicts a “one‐to‐many” relationship between sexual arousal and multiple biomarkers. In such a case, one cannot interpret the absence of genital arousal as the absence of “sexual arousal” more generally, because genital arousal is only *one* of the biological processes associated with the experience of arousal. Self‐reported arousal might diverge from genital blood flow while converging with *other* biological measures of arousal. Accordingly, we cannot necessarily characterize an individual as showing convergent *or* divergent patterns of response to sexual stimuli on the basis of any one of these measures in isolation. For example, studies have found that women show divergence between self‐reported arousal and SNS activity, paralleling the aforementioned findings regarding genital blood flow (Lorenz et al. 2012; Meston and Gorzalka 1996), but self‐reported arousal shows relatively greater convergence with vaginal lubrication and vulvar temperature (Bouchard et al. 2017; Dawson et al. 2015; Handy and Meston 2021; Huberman et al. 2017; Sawatsky et al. 2018). Accordingly, the same individual might show biodivergence with one biological measure, but not with others. Put simply, biodivergence diverges.

The lower left corner of Figure 1 brings other psychological states into the frame of analysis, and depicts a *many‐to‐one* relationship, in which a single biomarker of sexual arousal (in this case, levels of oxytocin) is associated with more than one psychological state. In such cases, convergence between the biomarker and a particular state might actually be attributable to another co‐occurring psychological state. Oxytocin provides a paradigmatic example because its release is known to be associated not only with sexual arousal, but *also* psychological stress *and* prosocial emotion (reviewed in Alley et al. 2019). Accordingly, we cannot interpret the relationship between oxytocin and any one of these states as “divergent” or “convergent” without understanding the presence or absence of co‐occurring states. Finally, the lower right quadrant shows the most accurate pattern of relations regarding biomarkers of sexual arousal: *Many‐to‐many* relations, in which multiple psychological states are associated with multiple biomarkers of sexual arousal. Because of this multiplicity, the presence or absence of correlations between *any* two domains is impossible to globally characterize as either convergent or divergent.

### 1.4 Self‐Report Is Multiple, Too

Figure 1 depicts multiplicity in biomarkers of sexual arousal, while representing the state of self‐reported sexual arousal as singular and coherent. Yet research shows that self‐reported sexual arousal is *also* a complex and multiply determined phenomenon, further complicating our interpretations of biodivergence. The simple fact that self‐reported sexual arousal *must be reported* introduces sources of cognitive, attentional, and motivational variability that might additively and interactively affect convergence with different biological processes. So far, no single overarching pattern has emerged from studies attempting to parse motivational influences on self‐reported arousal: For example, neither social desirability, impression management, nor sexual inhibition has been found to moderate divergence between women's biological and self‐reported arousal to laboratory stimuli (Boyer et al. 2012; Brody et al. 2003; Clifton et al. 2015; Velten et al. 2016). Interoceptive awareness, however, may play a role: Women who report greater abilities to perceive and interpret internal bodily signals tend to show greater convergence between biological and self‐reported arousal, especially when asked to specifically focus on bodily cues (Dorn et al. 2019; Handy and Meston 2016).

Another factor to consider is women's conscious and unconscious sexual memories and associations. Chivers and Blumenstock (2024) recently advanced a bold new interpretation of between‐person variation in divergence between self‐reported and biological measures of sexual arousal. Specifically, they called attention to the (often overlooked) fact that laboratory studies typically find greater bio‐divergence among heterosexual‐identified women than queer‐identified women. They argue against viewing this as a “sexual orientation effect” within women, and instead claim it might actually represent a *sexual experience and conditioning effect*. Specifically, they suggested that women's lifetime histories of pleasure and orgasm with different genders and sexual behaviors might provide an additional influence on their patterns of laboratory responses to sexual stimuli, because of straightforward learning and conditioning processes. They reviewed extensive research showing that heterosexual‐identified women are less likely than queer‐identified women to have had consistent experiences of pleasure and orgasm across prior experiences of sexual behavior with their “preferred” partners. As a result, heterosexual women's initial preferences for a certain gender (men) may not line up with their body's *history of sexual reward* with that gender, resulting in less consistent sexual responses to such stimuli (Chivers and Blumenstock 2024).

They support this claim with a convincing review of studies indicating that individuals' genital responses to sexual stimuli in the laboratory cannot be interpreted as tidy windows into automatic sexual processing but *also* incorporate learned responses to previous experiences. As a result, we cannot conceive of sexual responses as indicating any one “thing” about a person's sexuality—their triggers and drivers are inherently multiple. This makes it difficult for researchers to even *classify* sexual stimuli as “preferred” or “non‐preferred,” on the sole basis of a participant's self‐described orientation (heterosexual, bisexual, lesbian). After all, once we acknowledge a role for learning and conditioning processes in women's biological processes of arousal, we must reckon with the awareness that we may never know for certain what might be eliciting one form of biological response versus another. As Chivers and Blumenstock cautioned, “defining what is preferred is tricky. Is it the gender of the people depicted? The relationship context? The sex acts? The intimacy depicted?” (2024, 86).

It could be each of these. And more. In essence, Chivers and Blumenstock (2024) showed that the biological responses to sexual stimuli that researchers have historically interpreted as singular indices of orientation were never singular indices of *anything*. They were, and are, necessarily engaging multiple intersecting preferences, memories, expectations, and learned responses, along with a host of other potential meanings that may be outside the participant's *and* the researcher's conscious awareness (or capacity to assess). Furthermore, each of these multiple “triggers” within a single sexual stimulus might interact differently with different forms of biological responsiveness.

In light of this complexity, it seems impossible to sustain a notion of sexual orientation as a unitary, coherent phenotype—it, too, is better conceived as a set of patterns. And in fact, this is exactly what new genetic evidence on sexual phenotypes seems to indicate.

### 1.5 Divergence at the Level of Genotype

As I have reviewed elsewhere (Diamond 2021), genome‐wide association studies have provided some of the most powerful evidence that sexual orientation is not one phenotype, but many. Ganna et al. (2019) conducted the largest and most reliable genetic study of same‐gender sexuality to date, analyzing the complete genomes of 477 522 US and UK citizens, 4% of whom reported having at least one same‐gender sexual partner over their lifetime. Their analyses focused on predicting an individual's *lifetime number of same‐gender partners*. They acknowledge that this outcome cannot be interpreted as a proxy for “sexual orientation,” given that sexual behavior is driven by both motivational and opportunistic factors, yet it was the only outcome for which they had consistent data across all the data sources they used.

Ganna and colleagues' primary analysis sought to identify genetic predictors of *ever* having engaged in same‐gender sexual contact versus *never* having done so. The results indicated significant genetic influence on this outcome: approximately 8%–25% of the population variance in this outcome could be attributed to genetic differences between individuals. Yet importantly, no single gene was responsible. Rather, they found that *thousands* of genetic variants, each with tiny effects, collectively contributed to the likelihood that an individual “ever” versus “never” pursued same‐gender behavior. Furthermore, they emphasized that the magnitude of each genetic influence was so small that there was no meaningful way to reliably predict any individual's status (“ever” versus “never”) based on their genome. Earlier attempts to identify genetic markers for sexual orientation sought *singular* loci associated with sexual orientation, but these efforts were unsuccessful (see Mustanski et al. 2005). Now we know why: there are *thousands* of genes related to the probability of pursuing same‐gender behavior, and even when we consider them simultaneously, their individual influences are too small to yield any meaningful predictions about a person's sexual patterning. In other words, sexual orientation is not a single phenotype with multiple genetic influences, but *multiple* phenotypes with *multiple* genetic influences.

The multiplicity of sexual orientation, at the level of both phenotype and genotype, finds even stronger support in Ganna et al.'s second set of analyses, which focused on predicting the proportion an individual's lifetime sexual partnerships pursued with same‐gender versus other‐gender partners (among those reporting any same‐gender contact). These analyses sought to test whether individuals with “exclusively gay” versus “bisexual” behavioral phenotypes were genetically distinct from one another. They were: In fact, they estimated that 27% of the variance in “proportion of same‐gender behavior” was heritable. Given that this heritability estimate is similar to the heritability estimate for “ever engaging in same‐gender behavior,” one might imagine that the same set of genes underlies both outcomes. Yet this was not the case: Ganna et al. found that the genes which predicted “proportion of same‐gender partners” were different from the genes which predicted “ever‐vs‐never had same‐gender contact.”

This unexpected pattern led Ganna et al. to conclude that there was no genetic basis for the well‐known Kinsey scale, which arrays sexual orientations along a single continuum ranging from exclusive opposite‐gender behavior (0) to exclusive same‐gender behavior (6), with bisexual patterns in between (1–5). If there were a single genetic continuum ranging from exclusive heterosexuality to exclusive same‐gender sexuality, then the same genes that distinguished between individuals who “ever” versus “never” pursued same‐gender contact should also distinguish among individuals (*within* the “ever” group) who pursued more versus less of that contact over their lifespans. In other words, the genes associated with being “not straight” should also be associated with one's degree of “not straightness.” Yet Ganna et al.'s results upend this intuitively‐appealing assumption. Rather, their results suggested that an individual's placement on the Kinsey scale (i.e., their self‐perceived orientation) represents *two* genetic traits: The first relates to *any* expression of same‐gender interest (Kinsey 0 versus all else); the second relates to degrees of same‐gender interest (Kinsey 1–6).

I have speculated elsewhere that perhaps the genes associated with “ever vs. never” engaging in same‐gender behavior have nothing to do with sexual orientation at all: Perhaps instead they are associated with a general predisposition for risk tolerance, or openness to experience. Some degree of risk tolerance may be necessary to facilitate acting on *any* same‐gender interests, but is independent of one's *degree* of same‐gender interest. This interpretation is consistent with the results of additional analyses conducted by Ganna et al. (2019) which examined how each of these traits related to *other* genetically‐influenced traits. They found that the genes associated with “ever vs. never” engaging in same‐gender contact were correlated positively with the genes associated with total number of lifetime sexual partners, openness to experience, cannabis use, smoking, and general risk behavior, whereas the genes associated with “proportion of same‐gender partners” showed the *opposite* pattern of correlations. This supports the notion that the genes associated with “ever vs. never” are more closely related to generalized risk behavior than to sexual orientation. Yet if Ganna et al. had not done a second set of genetic analyses with the “proportion of same‐gender partners” variable, *this might not have been evident*, and they might have inadvertently concluded that the genes associated with “ever vs. never” were specifically related to same‐gender sexuality. This underscores the danger of interpreting convergence or divergence involving any *single* biological measure, when the phenomenon of interest is inherently multiple.

### 1.6 Multiple Levels of Multiplicity

**FIGURE 2:**
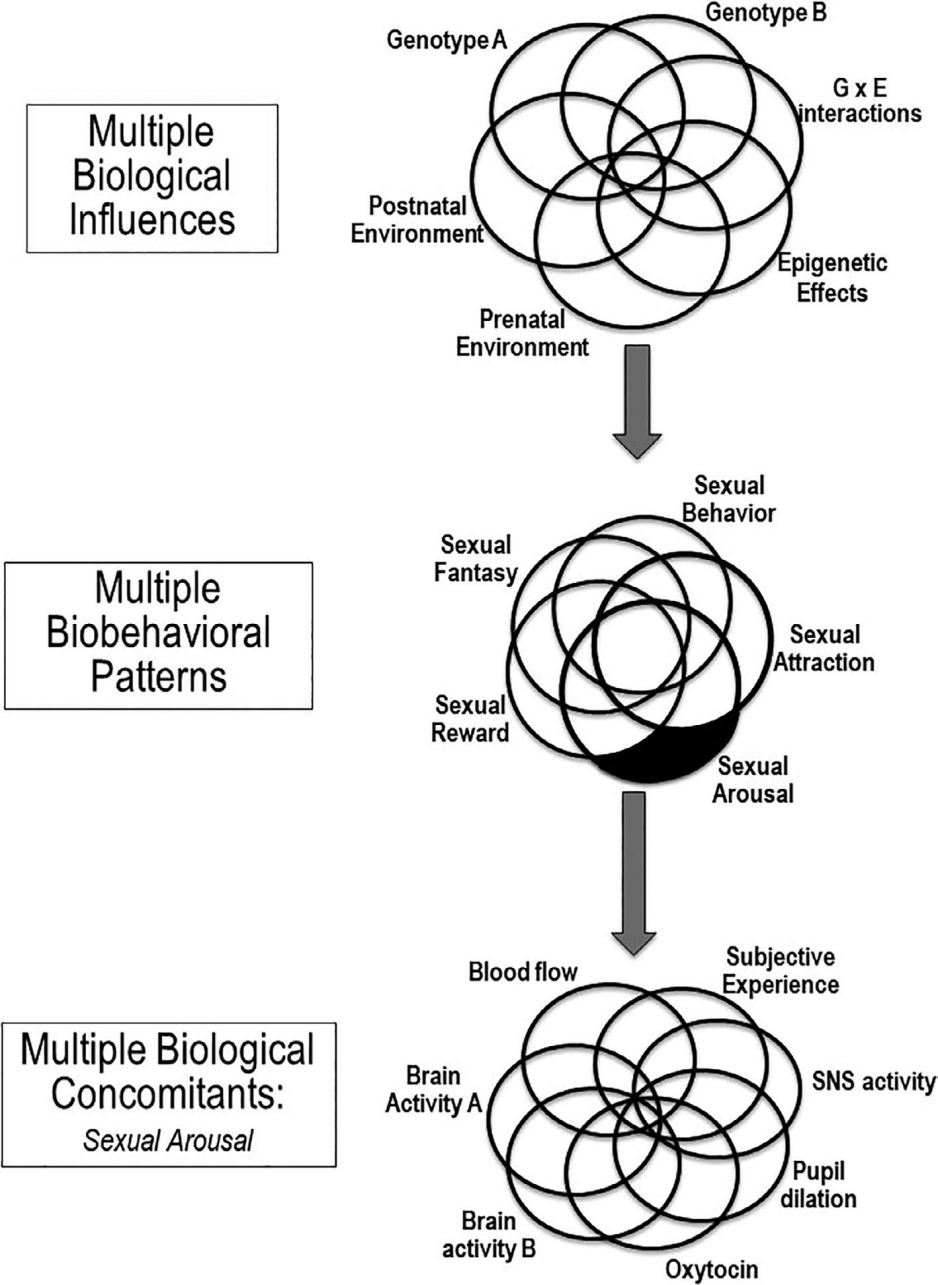
Multiple biosocial influences on, manifestations of, and biological concomitants of “sexual orientation”.

The results of Ganna et al.'s (2019) study provide the strongest possible corrective to the notion of sexual orientation as a singular phenotype with a singular set of genetic influences. What I find particularly useful about their findings is that they provide a critique of biological essentialism and biological reductionism *that is based squarely in biology*. Often, variability and multiplicity in the expression of sexual diversity around the globe is attributed solely (or primarily) to cultural variation; but Ganna et al.'s findings show that such multiplicity extends to the biological level. Figure 2 provides a graphical representation of this multiplicity: The top part of the figure uses overlapping circles to represent the various potential influences on human sexual orientation that have been studied over the past several decades: As reviewed by VanderLaan et al. (2022), there is partial—and often conflicting—evidence for *each* of these potential contributors. But what exactly are they contributing *to*? The middle panel of the figure depicts the various “trait manifestations” of sexual orientation that have been studied over the past several decades, and which have shown consistently inconsistent relations with one another. Numerous scholars over the years have documented divergence between different facets of sexual personhood (identity versus behavior, behavior versus attraction, attraction versus identity, fantasy versus behavior), and there is no consensus on which of these represents the “core” manifestation of sexual orientation (reviewed in Diamond 2008). In Figure 2, I have shaded “sexual arousal” in black to call attention to the fact that although *this* is the manifestation of sexual orientation which has been extensively studied in laboratory research (and for which biodivergence has been reliably observed, as reviewed above), it is only *one* of the many different potential trait manifestations of sexual orientation, and there is no scientific consensus on which of these manifestations should be considered “primary.”

The bottom panel of the figure “zooms in” to focus specifically on the multiple biological contributors and concomitants of sexual arousal, which may or may not relate to one another (as reviewed above) *or* to the other “trait manifestations” of sexual orientation. When taken in sum, this figure demonstrates why we should never have been surprised to observe divergence at *any* of these levels of analysis. Although we have become accustomed to speaking of “sexual orientation” as a singular and coherent trait, there is no way to sustain this perspective once we fully account for the complex “many‐to‐many” relations that have been observed at every level of this phenomenon.

An additional complication is *time* (Diamond 2008, 2016). Prospective studies show that some degree of longitudinal change in sexual identity and expression is normative, rather than exceptional, and this has significant implications for interpreting the results of biological studies of sexual orientation. Consider Ganna et al.'s (2019) study: What might happen if they followed up with these 477 522 individuals in 5–10 years? Their genomes would be the same, but their lifetime ratios of same‐gender to other‐gender behavior might change quite a bit. As a result, it is possible that many of the genetic correlations reported in the study would change if they were re‐run with updated sexual behavior data in 5, 10, or 15 years. Which set of results is correct? Notably, only two studies have assessed longitudinal change in concordance (or lack thereof) between cisgender women's biological and self‐reported arousal to erotic stimuli: One of these studies found that women's degree of biodivergence remained stable over a 3‐month period (Velten et al. 2018), whereas the other found that it changed (Suschinsky and Lalumiere 2011). Once again, biodivergence diverges.

Given that some degree of dynamic change may be a robust feature of sexual expression and identification over the life course (Diamond 2007), one might argue that we cannot meaningfully interpret the presence or absence of biodivergence across *any* biological or self‐reported measures of sexuality from data collected during only one time point as a universal, cross‐situational “truth” (and Cacioppo and Tassinary 1990, include *responses over time* as an additional element requiring conceptualization in models of complex biopsychological relations). Not only might individuals' self‐reports change, but their biological parameters might change, given the extensive evidence for social and contextual influences on multiple biological processes and parameters (van Anders 2024). Accordingly, biologically‐oriented studies of sexual orientation should *begin* with the presumption of dynamic, bidirectional change in *both* biological and self‐reported parameters, and design their study protocols and analytical plans accordingly. For example, if we choose to examine discrepancies among different aspects of sexuality as outcomes in their own right, we must account for the possibility that these discrepancies *themselves* will show dynamic change, sometimes driven by one parameter and sometimes driven by another. One might argue that the *most* important form of biodivergence to study, in biobehavioral research on sexuality, is that between *any* aspect of sexuality at Time 1 and the same aspect at Time 1 + *x*.

## 2 Conclusion

Given the complexity of relations between psychological states and biological processes, biodivergence in studies of sexual arousal and orientation should not surprise us, and in fact similar findings of biodivergence are endemic within the broader field of psychophysiology (Cacioppo et al. 2003; Mauss et al. 2005). Cacioppo and Berntson (1992) have argued that although biodivergence sometimes stems from weaknesses in methodology and study design (Cacioppo et al. 2003), in other cases it signals a fundamental *theoretical* error, most commonly the presumption of singular rather than multiple causes and effects.

This, I would argue, is the error that has been laid bare by the past three decades' worth of experimental studies documenting divergence between individuals' biological and self‐reported responses to sexual stimuli. This body of work began with the presumption that sexual orientation represented a singular, coherent trait whose “output” was a singular, coherent pattern of sexual response. Yet these presumptions turned out not to be true. One might view the extensive findings on bio‐divergence in sexual arousal as necessary consequences of what Mol has called “the body multiple” (Mol 2002), an organizing framework for thinking through the inherent contradictions presented by the body's various and intersecting signals, processes, behaviors, and functions. Mol argued that the coherence we impose on these conflicting signals is illusory: When we observe divergent measures of the same “thing” (in this case, sexual arousal and/or orientation), it is because we are observing divergent *things*. The multiplicity we observe is not a sign of measurement error or distortion; it is inherent.

From this perspective, we should steadfastly resist the temptation to try and resolve divergence between biological measures of the “trait” of sexual orientation or the “state” of sexual arousal, but instead view these as *different, multiply‐determined phenomena*. Hence, although scientists have historically conceived of “sexual arousal” as the coherent, oriented biopsychological experience lying at the heart of an individual's sexual phenotype, this perception is illusory, because neither arousal nor orientation is singular nor coherent. We may *perceive* some degree of coherence in our own experiences of arousal, or in our subjective experience of “orientation/identity,” but this perception is akin to perceiving a cluster of yellow and blue dots as uniformly *green*. Following Mol's logic, there are as many “states” of sexual arousal as there are modalities for experiencing and measuring it (brain activation, genital blood flow, respiratory rate), and the convergence among any of these may be situationally contingent.

Western culture has a long history of “naturalizing” social divisions by linking them to biological processes (Choderow 1978; Jordan‐Young 2011). Given our culture's privileging of biological knowledge about human experience, the use of biology for “carving nature at its joints” (VanderLaan et al. 2022) is intuitively appealing. Yet when we use biology to discern boundaries between observed human groupings (child—adult, homosexual—heterosexual, male—female, white—nonwhite, etc.), we risk obscuring our own active role in this process. Humans are never passive observers of human categories—whether we deploy biology or self‐report, we are always making “agentic cuts” (Barad 2007) that actively carve bounded categories out of complex systems, based on our own socially situated expectations, observations, and assumptions. As a result, biologically based groupings can be just as biased as those based on self‐report or on behavior.

The “truth” about human sexuality resides neither in our cells nor our words nor our behaviors, but some dynamic relation among them all. van Anders (2024) argued that we should actively counter biological reductionism with *biological expansiveness/emergence*: “emergence refers to phenomena that cannot be predicted by their constituent parts, akin to gestalts or ‘the whole is more than the sum of its parts.’ Expansiveness reflects how biological factors can expand the complexity of our work rather than simplify it, lead to new and dynamic questions rather than resolve them, and opens up space in myriad ways” (p. 482).

We will need this new space, and these new questions, to understand the potentially novel sexual and gender phenotypes that have emerged in the youngest generation of sexually diverse and gender diverse individuals (reviewed in Hammack 2025) and to make sense of their differences from previous cohorts without resorting to hierarchies of alignment which privilege concordant patterns of sexual expression over discordant patterns. The phenomenon of sexual diversity must be defined to include not just different sexual phenotypes, but different within‐person relations among drivers and determinants of these phenotypes across the life course.

## Funding

The author has nothing to report.

## Ethics Statement

The author has nothing to report.

## Data Availability

Data sharing not applicable to this article as no datasets were generated or analyzed during the current study.
